# Cullin-RING ligases in regulation of autophagy

**DOI:** 10.1186/s13008-016-0022-5

**Published:** 2016-06-10

**Authors:** Danrui Cui, Xiufang Xiong, Yongchao Zhao

**Affiliations:** Key Laboratory of Combined Multi-organ Transplantation, Ministry of Public Health, the First Affiliated Hospital, Zhejiang University School of Medicine, 79 Qing-Chun Road, Hangzhou, Zhejiang 310003 People’s Republic of China; Institute of Translational Medicine, Zhejiang University School of Medicine, 268 Kai-Xuan Road, Hangzhou, Zhejiang 310029 People’s Republic of China

**Keywords:** CRL E3 ligase, UPS, Autophagy, mTOR, Ubiquitin, NEDD8, ATG

## Abstract

Cullin-RING ligases (CRLs), the largest E3 ubiquitin ligase family, promote ubiquitination and degradation of various cellular key regulators involved in a broad array of physiological and pathological processes, including cell cycle progression, signal transduction, transcription, cardiomyopathy, and tumorigenesis. Autophagy, an intracellular catabolic reaction that delivers cytoplasmic components to lysosomes for degradation, is crucial for cellular metabolism and homeostasis. The dysfunction of autophagy has been proved to associate with a variety of human diseases. Recent evidences revealed the emerging roles of CRLs in the regulation of autophagy. In this review, we will focus mainly on recent advances in our understandings of the regulation of autophagy by CRLs and the cross-talk between CRLs and autophagy, two degradation systems. We will also discuss the pathogenesis of human diseases associated with the dysregulation of CRLs and autophagy. Finally, we will discuss current efforts and future perspectives on basic and translational research on CRLs and autophagy.

## Background

The long-term health of a cell is closely associated with protein quality control which requires a well-regulated balance between protein synthesis and degradation [1]. It is critical for the maintenance of cellular homeostasis to eliminate unwanted and aberrant intracellular proteins, which is charged by both the ubiquitin–proteasome system (UPS) and the autophagy–lysosome system in a coordinated manner [2]. Thus, the dysregulation of UPS and autophagy disrupts cellular homeostasis and causes many human diseases, such as heart failure, neurodegeneration, and cancer [3, 4].

**Fig. 1:**
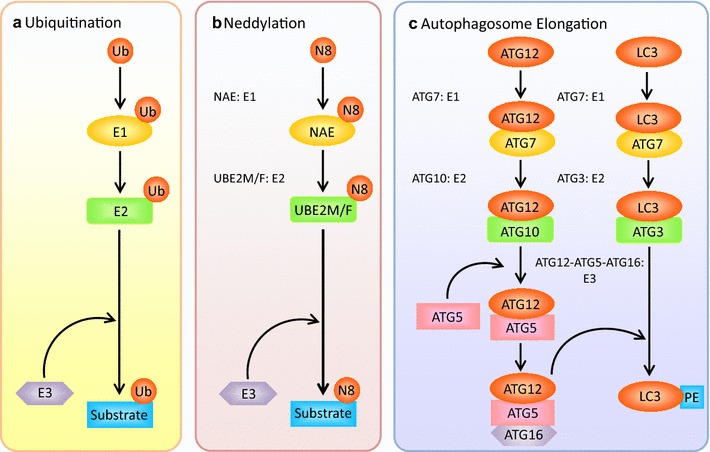
Ubiquitin system and ubiquitin-like systems. **a** The ubiquitination of substrates involves a three-step enzymatic reaction. **b** Neddylation, like ubiquitination, is a trio of enzymatic steps. **c** Two ubiquitin-like systems, ATG12-conjugation system and LC3-conjugation system, in autophagosome elongation

The UPS, a clearance system, directs target proteins with their lysine residues and the N-terminal methionine residue covalently attached by ubiquitin molecules, to the 26S proteasome for degradation, leading to the elimination of short-lived, misfolded, and damaged proteins [5–8]. Protein ubiquitination is a trio of enzymatic steps mediated by E1 (ubiquitin-activating enzyme), E2 (ubiquitin-conjugating enzyme), and E3 (substrate-specific ubiquitin ligase) [9]. First, ubiquitin is activated in an ATP-dependent reaction catalyzed by E1. Second, the activated ubiquitin is transferred to the active site of an E2. Finally, an E3, which recognizes and recruits the target protein, designated as substrate, mediates the transfer of the activated ubiquitin directly to a lysine residue on the substrate (Fig. 1a). Ubiquitin has seven lysine residues (K6, K11, K27, K29, K33, K48, and K63) and the N-terminal methionine residue, on which the poly-ubiquitin chains can be formed. The distinct fate of ubiquitinated proteins depends on the nature of ubiquitin attachment and the type of isopeptide linkage of the poly-ubiquitin chain. Target proteins marked with K48- or K11-linked poly-ubiquitin chain predominantly are recognized and degraded by the proteasome, whereas mono-ubiquitination and K63-linked polyubiquitination usually alter protein function and subcellular localization [10–12].

Autophagy is a highly conserved eukaryotic intracellular catabolic degradation process in which cytoplasmic contents, like misfolded proteins and damaged organelles, are engulfed by double-membrane autophagosomes and degraded in lysosomes fused with autophagosomes [13]. In general, autophagy is thought to be a nonselective degradation system, which is different from UPS by selectively targeting polyubiquitinated proteins for degradation [14]. There are generally three types of autophagy: macroautophagy, microautophagy, and chaperone-mediated autophagy (CMA) [15, 16]. Macroautophagy, generally termed “autophagy” unless specified, has received the greatest attention and is the best-characterized form of autophagy. Tons of evidences have shown that autophagy plays important roles in diverse biological processes, such as intracellular protein and organelle clearance, starvation adaptation, development, and tumorigenesis [17].

Autophagy, or “self-eating”, plays a vital role in the maintenance of cellular homeostasis. The self-digestion maintains critical physiological functions by providing nutrients during fasting and by eliminating the “garbage” in cells, such as aggregated proteins, damaged organelles, and invading pathogens [4]. Such functions are likely key to autophagy-mediated physiological and pathological processes as diverse as development, aging, immune response, neurodegeneration, heart failure, and cancer [4, 18]. However, the pro-survival functions of autophagy in certain disease settings may be deleterious. A good example is the dual role of autophagy in cancer progression [19]. On one hand, autophagy acts as a tumor suppressive mechanism through the elimination of aggregated proteins and damaged organelles. On the other hand, it is a key cell survival mechanism by which it facilitates the resistance of established tumors to radiation and chemotherapy. Therefore, the recognition of autophagy function might depend on the specific context.

Given the primary role of autophagy in cellular homeostasis, it is not surprising that the whole process is tightly controlled. Including phosphorylation, ubiquitination, and acetylation, multiple types of post-translational modifications have been found in the regulation of autophagy [20]. This review summarizes our current knowledge of the role of ubiquitination mainly mediated by CRLs in the regulation of autophagy. A thorough understanding of the cross-talk between CRLs and autophagy should lead to new insights into the development of novel therapy for associated diseases.

## General features of CRLs

Cullin-RING ligases (CRLs), the largest family of E3 ubiquitin ligases, account for ubiquitination of approximately 20 % cellular proteins degraded by UPS [21]. The following part will describe briefly main features of CRLs, including their composition, and dynamic regulation of CRL assembly and activation mainly mediated by neddylation.

### The composition of CRLs

**Fig. 2:**
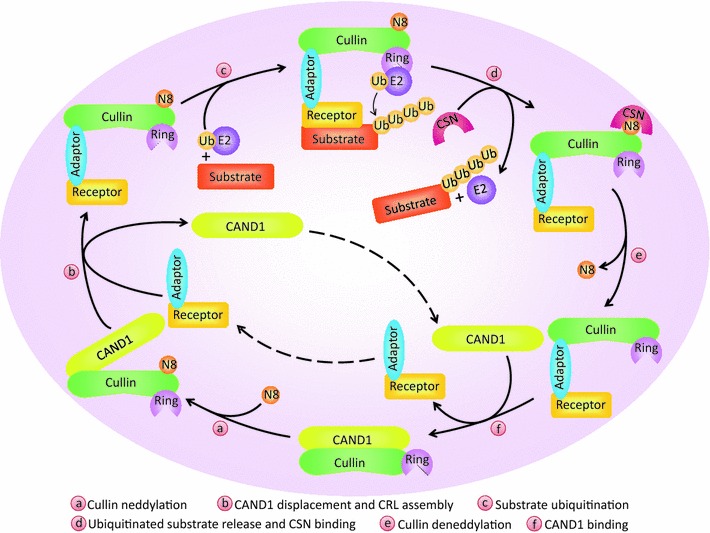
Dynamic regulation of CRLs activity by neddylation and deneddylation. CAND1 binding to unmodified cullin blocks the interaction of cullin with the substrate receptor-adaptor module. Cullin neddylation promotes the dissociation of cullin from CAND1 and restores the CRLs in an active conformation, leading to substrate ubiquitination. After the detachment of ubiquitinated substrate from CRLs, NEDD8 is removed by CSN from cullin for recycling. At last, CAND1 binds to cullin and inactivates CRLs

Generally, CRLs consist of four elements: cullins, RING-finger proteins, adaptor proteins, and substrate recognition receptors (Fig. 2). The human genome encodes 8 cullins (CUL1, 2, 3, 4A, 4B, 5, 7, and 9, also known as PARC), 2 RING-finger proteins (RBX1 and RBX2, also known as ROC1 and ROC2/SAG, respectively), 4 adaptor proteins (SKP1 for CUL1/7, Elongin B/C for CUL2/5, and DDB1 for CUL4A/B), and more than 400 substrate recognition receptors (69 F-box proteins for CRL1, 80 SOCS proteins for CRL2/5, about 180 BTB proteins for CRL3, and 90 DCAF proteins for CRL4A/B) [22–28]. Thus, at least 400 CRLs can be assembled in human cells and regulate diverse biological processes by targeted ubiquitination and degradation of thousands of substrates (for a recent review, see Ref. [23]).

All CRLs share the similar core architecture with a curved cullin protein acting as a molecular scaffold [22, 29]. Among all CRLs, CRL1, also known as SCF (SKP1-CUL1-F-box), is the most characterized member of CRLs [30]. CUL1 consists of three repeats of a five-helix motif at the N-terminus and a globular domain at the C-terminus. SKP1, the adaptor protein, and RBX1 or RBX2, a RING protein, bind to the N-terminus and the C-terminus of CUL1, respectively. Then, SKP1 binds to an F-box receptor, which specifically recognizes the substrate, whereas the RING protein binds to ubiquitin-charged E2 and effectively catalyzes the transfer of ubiquitin from E2 to the specific substrate [29, 30]. It is well established that the core E3 ligase activity is possessed by the CUL1-RBX1/2 complex in which the RING finger domain of RBX1/2 binds to two zinc atoms via a C3H2C3 motif, and that the substrate specificity of SCF is determined by F-box receptors [24, 31]. Moreover, all cullins contain an evolutionarily conserved lysine residue at its C-terminus for targeted NEDD8 modification, a reaction known as neddylation, which is vital to CRLs activation [32].

### The regulation of CRLs activity by neddylation

Neddylation, like ubiquitination, is a process of the attachment of ubiquitin-like molecule NEDD8 to target proteins, involving the successive actions of E1 NEDD8-activating enzyme (a heterodimer of APPBP1/UBA3, also known as NAE), which activates NEDD8; E2 NEDD8-conjugating enzyme (UBE2M, also known as UBC12, or UBE2F), which carries the activated NEDD8; and E3 NEDD8 ligase, which recognizes the substrate and catalyzes the transfer of NEDD8 from E2 to the substrate [33] (Fig. 1b).

An impressive feature of CRLs is that their activity is dynamically regulated by neddylation and deneddylation. The binding of unmodified cullins to CAND1 (cullin-associated and neddylation-dissociated 1) blocks the interaction of the substrate receptor-adaptor complex with the N-terminus of cullins. However, covalent conjugation of cullin with one NEDD8 molecule removes the inhibitory binding to CAND1 and restores the CRLs in an active conformation [34–36]. In addition, neddylation enhances and stabilizes the recruitment of ubiquitin-loaded E2 to CRLs, facilitates the initial ubiquitin transfer and also increases the elongation rate of poly-ubiquitin chain [37–40]. After dissociation of polyubiquitinated substrate from CRLs, NEDD8 is detached by the COP9 signalosome complex (CSN) from cullins for recycling, a reaction known as deneddylation [41] (Fig. 2). The activation cycle of CRLs by dynamic neddylation and deneddylation is essential for the maintenance of cellular homeostasis. Moreover, this cycle assists the recycling of the cullin-RING core that will make it possible for the assembly of other CRLs to allow the ubiquitination of various different substrates as required by the cells in a short time [42]. On the other hand, the inactivation of all CRLs can be achieved by inhibiting cullin neddylation. Indeed, MLN4924, a newly discovered inhibitor of NAE, blocks the entire neddylation pathway, and thus serves as an indirect inhibitor of CRLs [21]. Treatment of MLN4924 causes the accumulation of a number of CRLs substrates and consequently induces cell apoptosis, senescence, and autophagy [43], suggesting that CRLs may regulate autophagy.

## Basic concepts of autophagy

### Core machinery of autophagy

**Fig. 3:**
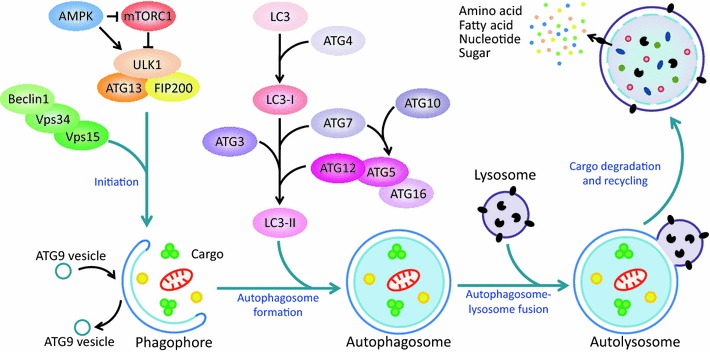
A schematic summary of autophagy process and core machineries in autophagosome formation. Autophagy, a sequential process, consists of initiation, autophagosome formation, autophagosome–lysosome fusion, and cargo degradation, followed by recycling of macromolecules. This process is precisely regulated by different sets of ATGs and involves at least four core machineries, including the ULK1 complex, the Beclin-1/Class III PI3K complex, ATG9 and its recycling system, and two ubiquitin-like protein conjugation systems

In mammalian cells, autophagy consists of several sequential steps: initiation, autophagosome formation, cargo recognition and delivery, autophagosome–lysosome fusion, and cargo degradation followed by recycling of the resulting macromolecules via permeases, all of which are coordinated by different sets of ATGs (Fig. 3). Among these steps, autophagosome formation is the key process in autophagy, which is regulated by at least four complexes, known as the core machinery of autophagy, including the ULK1 (a homologue of yeast Atg1) complex, the Beclin-1/Class III PI3K complex, ATG9 and its recycling system, and two ubiquitin-like protein conjugation systems [44–46] (Fig. 3).

The activity of the ULK1 complex (along with ATG13 and FIP200) is required for the autophagy induction. Under physiological conditions, the activated mTORC1 directly binds to the ULK1 complex and inhibits autophagy by phosphorylating ULK1 on Ser757 and ATG13 [47, 48]. Under unfavorable conditions, mTORC1 is inactivated and disconnects from the ULK1 complex. ULK1 is then auto-phosphorylated and then phosphorylates ATG13 and FIP200. As a result, the whole ULK1 complex is activated [49–51], which translocates to ER or other specific places to help with the nucleation of autophagosome formation, followed by the recruitment of downstream effectors including the Beclin-1/Class III PI3K complex and LC3 (a homologue of yeast Atg8) to the site where the nucleation takes place [52].

The Beclin-1/Class III PI3K complex, consisting of Beclin-1, Vps34, and Vps15, is essential for the nucleation of phagophore [53]. It catalyzes the phosphorylation of phosphoinositide to produce phosphatidylinositol-3-phosphate (PI3P), which recruits those effectors containing PX or FYVE domain (such as DFCP1 and WIPI) to mediate the formation of the initial sequestering vesicle (also known as phagophore) that develops into the autophagosome [54, 55]. Accumulating evidences revealed that Beclin-1 serves as an adaptor to recruit multiple proteins, such as ATG14, Ambra1, UVRAG, and Rubicon [56], that modulate the kinase activity of Vps34. Additionally, Beclin-1 is highly regulated in autophagy by post-translational modifications, including phosphorylation, ubiquitination, and cleavage [57].

ATG9, a multipass transmembrane protein, is essential for autophagosome formation. ATG9 is localized to the trans-Golgi network (TGN) and late endosomes. Following the induction of autophagy, ATG9 is rearranged from juxtanuclear to peripheral structures, and is then retrieved from the completed autophagosome [58]. Thus, ATG9 recycling delivers the membrane to the forming autophagosome. Both the ULK1 complex and the Beclin-1/Class III PI3K complex are involved in regulating this process [46, 59].

The elongation and expansion of the phagophore membrane is controlled by two ubiquitin-like conjugation systems. First, the E1-like enzyme ATG7 and the E2-like enzyme ATG10 mediate the covalent conjugation of ATG12 to ATG5 [60]. The resulting ATG5-ATG12 noncovalently interacts with ATG16 to form the ATG12-ATG5-ATG16 complex, which acts as the E3 ligase towards LC3. Next, the E1-like enzyme ATG7 and the E2-like enzyme ATG3 sequentially act to conjugate phosphatidylethanolamine (PE) to a glycine residue of LC3, which constitutes the other conjugation system with ATG12-ATG5-ATG16 complex, the E3-like enzyme [61]. This process achieves the conversion of LC3-I, the soluble form of LC3, to LC3-II (also known as LC3-PE), the autophagic vesicle-associated form (Figs. 1c, 3). In addition, it is worth noting that ATG4, a cysteine protease, plays critical roles in the proteolysis of the full-length isoform of LC3 (pro-LC3), as well as in the deconjugation of the lipidated LC3-PE for recycling [62, 63].

### Signals that regulate autophagy

Autophagy is induced by a range of cellular stresses, including nutrient and energy depletion, ER stress, hypoxia, redox stress, and oncogenic activation [64]. It is regulated either negatively or positively by the following two biologically significant molecules.

**Fig. 4:**
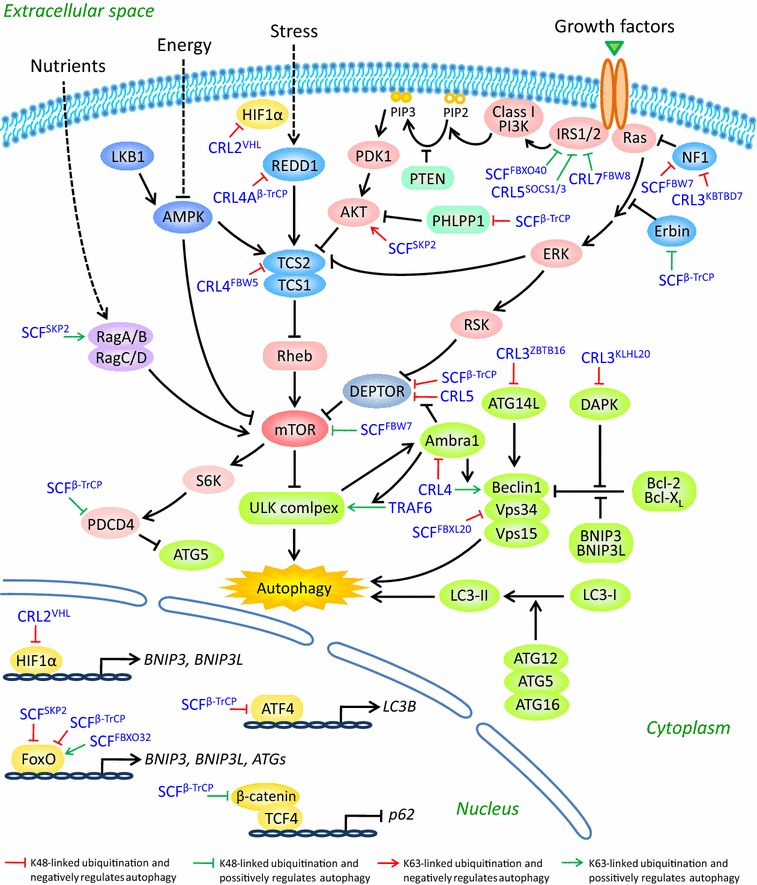
A schematic summary of CRLs substrates in the regulation of autophagy. CRLs control autophagy at multiple levels. First, CRLs mediate the ubiquitination of several components of autophagy machinery. Second, CRLs regulate the activation of mTOR pathway, the central regulator of autophagy. At last, several key transcription factors involved in autophagy are also the substrates of CRLs. See *text* for details

mTOR is the well-established negative regulator of autophagy. It plays a key role in the coordination of cell growth with autophagy in response to physiological and environmental conditions [65]. mTOR, an evolutionarily conserved serine/threonine protein kinase, forms two structurally and functionally distinct complexes (namely mTORC1 and mTORC2) in mammalian cells. mTORC1 is composed of mTOR, raptor, PRAS40, mLST8, and DEPTOR; mTORC2 also contains mTOR, mLST8, and DEPTOR, but instead of raptor and PRAS40, the proteins rictor, mSin1, and protor exclusively exist in mTORC2 [66]. Moreover, mTORC1, as a negative regulator of the ULK1 complex, inhibits autophagosome formation in response to diverse signals [65], whereas mTORC2 inhibits autophagy through repressing the transcription of some *ATGs* via AKT-FoxO3 signaling [67, 68] (Figs. 3, 4).

AMP-activated protein kinase (AMPK), a master regulator of energy metabolism, is a vital positive regulator of autophagy. As a serine/threonine kinase, AMPK is activated when the levels of AMP and ADP in the cells rise owing to various physiological stresses [69]. Upon phosphorylated by activated AMPK, a range of substrates not only acutely affect metabolism and growth, but also are responsible for the long-term metabolic reprogramming. AMPK induces autophagy through phosphorylation of TSC2 and raptor to inhibit mTORC1 [70, 71], and through ULK1 phosphorylation on Ser317 and Ser777 to activate ULK1 [47].

Taken together, by integrating both intracellular and extracellular signals, mTOR and AMPK function coordinately in the regulation of autophagy (Figs. 3, 4).

## The role of CRLs in the regulation of autophagy

More recently, the study on the roles of post-translational modifications in regulation of autophagic flux by affecting the activity, recruitment, and turnover of autophagic components has become an attractive area due to the implications of dysregulated autophagy in multiple diseases [20]. Ubiquitination, an important cellular post-translational modification, plays a principal role in controlling protein turnover, activation, subcellular localization, and protein–protein interactions. However, current knowledge of the roles of E3 ubiquitin ligases in the regulation of autophagy is fairly limited. It was reported that RNF5, a RING finger E3 ligase, negatively regulates autophagy by controlling the stability of ATG4B [72], and that Parkin, a RING-HECT hybrid E3 ligase, induces mitophagy through ubiquitination of multiple mitochondrial proteins [73, 74]. Furthermore, the role of CRLs, the largest E3 ubiquitin ligase family, in regulating autophagy is rarely mentioned. Here, we discuss the emerging roles of CRLs in the control of autophagy, especially those regulating autophagy machinery and upstream regulators.

### CRLs regulate autophagy machinery

Several components of autophagy machinery are subjected to CRLs-mediated regulation. For instance, SCF^FBXL20^ targets Vps34, the catalytic subunit of the Beclin-1/Class III PI3K complex, for ubiquitination and proteasome degradation, and plays an important role in DNA damage-induced suppression of autophagy [75, 76]. Meanwhile, ATG14L (Atg14 in yeast), a crucial player to initiate autophagosome formation by mediating the production of PI3P, is the substrate of CRL3^ZBTB16^. Thus, CRL3^ZBTB16^ controls the initiation of autophagy by regulating the degradation of ATG14L [77]. In addition, Beclin-1, an adaptor protein in the Beclin-1/Class III PI3K complex, can be modified with multiple poly-ubiquitin chains catalyzed by distinct E3 ligases [78–80]. Specifically, CRL4^Ambra1^ mediates K63-linked polyubiquitination of Beclin-1 and enhances its association with Vps34 to promote the activation of Vps34, which is required for starvation-induced autophagy [78]. Notably, Ambra1 (activating molecule in Beclin-1-regulated autophagy), also identified as DCAF3 (DDB1 and cullin4 associated factor 3), has multiple roles in the regulation of autophagy. First of all, Ambra1 acts as a substrate receptor for Beclin-1 ubiquitination [78]. Second, Ambra1 recruits the E3 ligase TRAF6 to promote K63-linked polyubiquitination of ULK1, resulting in the stabilization and activation of ULK1 [81]. Third, by dynamically interacting with CRL5 and CRL4, Ambra1 temporally controls the onset and the termination of autophagy response to stress [82]. Under unstressed conditions, Ambra1 is maintained at low levels through degradation by cullin4-DDB1, and autophagy is off. In the early starvation, Ambra1 is detached from cullin4-DDB1 and phosphorylated by ULK1. Thus, transiently stabilized Ambra1 binds to cullin5 to inhibit CRL5-mediated DEPTOR degradation (see below for details), leading to the suppression of mTOR activity. As a result, autophagy is on. In the prolonged starvation, cullin4-DDB1 associates with Ambra1 and targets its polyubiquitination and degradation, leading to the termination of autophagy [82, 83]. Altogether, Ambra1, as a bridge between CRLs and autophagy, plays vital roles in the regulation of autophagic flux at different stages in response to stress. Additionally, CRL3^KLHL20^ regulates IFN-induced autophagic death by the destruction of DAPK, a serine/threonine protein kinase, which is in charge of the dissociation of Beclin-1 from its Bcl-2 inhibitors by phosphorylating Beclin-1 on Thr119 located at a key position within its BH3 domain required for the interaction with Bcl-2 family members [84–86] (Fig. 4).

To date, the studies on the roles of CRLs in the regulation of autophagy machinery mainly focus on the ULK1 complex and the Beclin-1/Class III PI3K complex, both of which function in the early stage of autophagy. The roles of CRLs in other components of autophagy are fairly limited. Given that the whole process of autophagy is precisely coordinated, it will be intriguing and helpful to elucidate the roles of CRLs in regulating other autophagy machineries, such as ATG9 and its recycling system and two ubiquitin-like protein conjugation systems.

### CRLs regulate the upstream regulators of autophagy

Since mTOR pathway is a central regulator of autophagy, it is conceivable that CRLs regulate autophagy via modifying a variety of components, both upstream and downstream, of mTOR pathway [87]. First of all, mTOR itself was identified as a substrate of SCF^FBW7^ that negatively regulates mTOR protein stability [88]. Second, DEPTOR, a direct mTOR inhibitor, was reported to undergo ubiquitin-mediated degradation by SCF^β-TrCP^ on phosphorylation by the setting S6K1/RSK or mTOR/CK1 [87, 89–91], and was also proved recently to be a substrate of CRL5 [82]. Third, CRL4^FBW5^ controls TSC2 protein stability and the subsequent turnover of TSC complex, which is the major inhibitor of mTOR pathway [92]. Fourth, HIF1α, a negative regulator of mTORC1 via the REDD1-TSC1/2 axis, is a well-characterized substrate of CRL2^VHL^ [93, 94]. Fifth, REDD1, an inhibitor of mTORC1, was subjected to CRL4A^β-TrCP^-mediated ubiquitination and degradation with GSK-3β as a corresponding kinase for phosphorylation [95]. Sixth, PHLPP1, a protein phosphatase negatively regulating AKT via direct dephosphorylation of activated AKT, was identified as a substrate of SCF^β-TrCP^ in a manner dependent on CK1 and GSK3β [96]. Seventh, IRS1 and IRS2, as adaptor proteins to mediate insulin/insulin-like growth factor 1 signaling, are the substrates of CRL5^SOCS1/3^ [97]. IRS1 can also be ubiquitinated and degraded either by CRL7^FBW8^ upon prerequisite phosphorylation by mTOR and S6K [98] or by SCF^FBXO40^ [99]. Eighth, NF1, an inhibitor of Ras, was identified as a substrate of SCF^FBW7^ [100] and CRL3^KBTBD7^ [101]. Ninth, Erbin, an inhibitor of Ras-Raf signaling, is a newly discovered substrate of SCF^β-TrCP^, which targets Erbin for degradation to trigger autophagy by ROS accumulation [102]. At last, PDCD4, a downstream target of mTOR-S6K1 pathway, was reported to be a substrate of SCF^β-TrCP^ dependent on pre-phosphorylation at Ser67 by S6K1, and negatively regulates autophagy by inhibiting ATG5 protein expression [103, 104] (Fig. 4).

Therefore, given the fact that nearly all these CRLs substrates, except for mTOR and IRS1, are negative regulators of mTOR pathway, the general inhibition of CRLs would likely cause their accumulation to down-regulate mTOR pathway, leading to the induction of autophagy. Indeed, MLN4924, an indirect inhibitor of CRLs, induces autophagy in multiple cancer cell lines resulting from inactivating mTORC1 by the accumulation of DEPTOR and HIF1α [105]. Consistently, silencing of RBX1, one of two RING proteins in CRLs, also triggers autophagy response by the accumulation of DEPTOR [106]. All these findings suggest that modulation of the activity of CRLs regulates autophagy induction, which may provide a novel therapeutic strategy for autophagy-associated human diseases.

In addition, SCF^SKP2^ promotes K63-linked ubiquitination of RagA, which recruits GATOR1 to hydrolyze RagA^GTP^ and blocks mTORC1 lysosomal localization and activation, leading to autophagy induction [107]. Interestingly, AKT is also a nonproteolytic substrate of SCF^SKP2^. SCF^SKP2^ drives K63-linked ubiquitination of AKT, which is vital for ErbB-receptor-mediated AKT membrane recruitment and activation in response to EGF [108]. Notably, it is a paradox that, on one hand, SKP2-mediated RagA ubiquitination suppresses mTORC1 activation; on the other hand, SKP2 promotes ubiquitination of AKT and increases its activity, which further activates mTORC1. Thus, the function of SCF^SKP2^ in the regulation of autophagy depends on certain cell type and its context (Fig. 4).

Moreover, PHLPP1 not only triggers macroautophagy, but also regulates chaperone-mediated autophagy (CMA) [109, 110]. CMA selectively degrades cytosolic proteins delivered by a cytosolic chaperone in the lysosomes [15, 111]. PHLPP1 induces CMA through its inhibitory effect on AKT [110]. Given that PHLPP1 is a substrate of SCF^β-TrCP^, CRLs may regulate CMA by controlling PHLPP1 stability.

### CRLs regulate autophagy at the transcriptional level

In addition to regulating autophagy machinery and upstream regulators, CRLs also control autophagy at the transcriptional level through modification of several key transcription factors.

We already discussed that accumulation of HIF1α, as a well-established substrate of CRL2^VHL^, is partially responsible for MLN4924-induced autophagy [105]. In fact, besides via the HIF1α-REDD1-TSC axis to block mTORC1 activity, resulting in autophagy induction, HIF1α itself, as a transcription factor, could induce autophagy directly by transcriptional regulation of its target genes. In response to hypoxia, HIF1α is activated and promotes the transcription of *BNIP3* and *BNIP3L* (also known as *NIX*), both of which disrupt the Bcl-2/Beclin-1 complex, leading to the release of Beclin-1 from Bcl-2 and the subsequent induction of autophagy [112, 113]. In addition, NIX/BNIP3, also located at the outer membrane of mitochondria, contains a WXXL/WXXL-like motif that binds to LC3 and its homolog GABARAP, leading to mitophagy induction [114, 115]. Thus, CRLs may also regulate mitophagy by inducing the transcription of *NIX/BNIP3* via HIF1α.

Meanwhile, the transcription factor FoxO3 regulates autophagy in skeletal muscle by transactivating *NIX/BNIP3* [116]. In addition, FoxO factors (such as FoxO1 and FoxO3) induce autophagy by promoting the expression of multiple *ATG* genes, including *ATG4B*, *ATG8*, *ATG12*, *Vps34*, and *Beclin*-*1*, during muscle atrophy [116–118]. And cytosolic FoxO1 is also required for autophagy induction in a transcription-independent manner via the interaction of acetylated FoxO1 with ATG7 [119]. Given the critical role of FoxO factors in regulating autophagy, the specific CRLs in charge of their stability were identified. Both FoxO1 and FoxO3 were ubiquitinated and degraded by SCF^SKP2^ [120, 121]. Additionally, FoxO3 is also the substrate of SCF^β-TrCP^ in an IKKβ-dependent manner [122]. Moreover, Atrogin-1 (also known as MAFbx or FBXO32), as a muscle-specific F-box protein that forms a complex with SKP1-CUL1-RBX1, mediates K63-linked polyubiquitination and consequent transactivation of FoxO1/FoxO3 and is a central node in the regulation of autophagy during muscle atrophy [117, 123].

ATF4, a transcription factor induced by severe hypoxia and involved in the unfolded protein response (UPR), up-regulates LC3B by directly binding to its promoter to facilitate autophagy [124, 125]. ATF4, a short-lived protein with a half-life time of about 30 min, is degraded rapidly by proteasome, following SCF^β-TrCP^-mediated polyubiquitination [126]. More importantly, bortezomib, a potent inhibitor of the 26S proteasome, activates autophagy by proteasomal stabilization of ATF4 and ATF4-induced up-regulation of LC3B [124]. β-catenin, another well-known substrate of SCF^β-TrCP^, inhibits autophagosome formation by suppressing *p62* (also known as SQSTM1, an autophagy adaptor protein) expression via TCF4 [127]. Thus, SCF^β-TrCP^ paradoxically regulates autophagy through repressing LC3B or inducing p62, two key proteins in the process of autophagy (Fig. 4).

In conclusion, all these studies highlight the importance and complexity of CRLs in the regulation of autophagy. Given that 1) these findings are mostly associated with mTOR pathway, the ULK1 complex, and the Beclin-1/Class III PI3K complex; 2) one specific CRL can target various substrates; 3) the specific substrate is subjected to the regulation of multiple CRLs; 4) autophagy can be regulated at the transcriptional, translational, and post-translational levels, further studies should be directed to elucidate the functional network of CRLs in the whole process of autophagy.

## The effects of autophagy on CRLs and UPS

Accumulating evidences indicate that the active cross-talk exists between UPS and autophagy, two major intracellular clearance systems [2, 128, 129]. Inhibition of UPS enhances autophagic activity possibly as a compensatory mechanism [129, 130]. In contrast, long-term inhibition of autophagy has been shown to compromise the degradation of proteasomal substrates, which leads to the accumulation of short-lived regulatory proteins, particularly some oncoproteins, with predicted deleterious consequences [131]. For example, p62, a selective autophagy receptor for the ubiquitinated protein aggregates, is degraded by autophagy. Twist1, an oncogenic transcription factor, is polyubiquitinated by SCF^FBXL14^ and subsequently degraded by the proteasome [132]. However, accumulated p62 caused by autophagy deficiency binds to polyubiquitinated Twist1 and inhibits its proteasomal destruction, in consequence, promoting tumor cell growth and metastasis [133]. p62 abrogates the clearance of ubiquitinated short-lived proteins destined for proteasomal degradation through two possible manners: (1) p62 disrupts the binding of ubiquitinated proteins with their partners that escort them to the proteasome [131, 133]; (2) p62 together with proteasomal substrate forms oligomer, which would be too bulky to be degraded by the proteasome in its narrow catalytic pore [128]. Paradoxically, p62 was also reported to interact with ubiquitinated proteins and deliver them (such as Tau) to the proteasome for degradation [134, 135]. This discrepancy may be caused by diverse protein substrates, specific cellular context, and different cell types. Notably, p62 also can impair CRLs-mediated ubiquitination. Specifically, p62 was accumulated in autophagy-defective cells and interacts with Keap1 on the NRF2-binding site to disrupt the ubiquitination of NRF2 mediated by CRL3^Keap1^, resulting in the hyperactivation of NRF2, which may contribute to hepatoma development [136–138]. Moreover, ATG16L1, an essential component of the autophagosome, is necessary for the neddylation of CUL3 with unknown mechanism, which is required for the ligase activity of CRL3 [139]. Taken together, autophagy can adjust UPS via multiple mechanisms. Future studies to explore precise molecular mechanisms should facilitate the development of novel therapeutic strategies for autophagy-defective human diseases.

## CRLs and autophagy in diseases

Given the facts that UPS and autophagy are two cornerstones in the maintenance of cellular homeostasis, and CRLs are the largest E3 ligase family, it is conceivable that the dysfunction of CRLs and autophagy contributes to the pathogenesis of various human diseases. In this part, we will mainly discuss the diseases associated with the dysfunction of both CRLs and autophagy.

### 3-M syndrome

Genetic studies have demonstrated a crucial role of CUL7 E3 ligase in controlling growth. *CUL7* germline mutations, resulting in loss of its functional cullin domain, are responsible for 3-M syndrome, characterized by prenatal and postnatal growth retardation [140]. The cause of these growth defects with *CUL7* germline mutations may owe to the accumulation of CRL7 substrates. Indeed, IRS1, one of CRL7 substrates, was stabilized in *Cul7*^−/−^ MEFs with senescence phenotype. The increased IRS1 activates its downstream AKT and MEK/ERK pathways, both of which were shown to induce senescence [98]. This kind of senescence, also known as oncogene-induced senescence, is closely associated with development and tumorigenesis [141, 142]. Accumulating evidences revealed that autophagy facilitates oncogene-induced senescence [102, 143, 144]. Thus, the accumulation of IRS1 or other unknown substrate(s) of CRL7 may contribute to the senescence through affecting autophagic flux. Recently, CRL5 and CRL4 were found to control the onset and the termination of autophagy, respectively, by dynamically interacting with Ambra1 [82]. In fact, the data also showed that Ambra1 could bind to CUL7. However, the underlying physiological functions are not further explored [82]. These may offer one potential hint that CUL7-mediated autophagy by interacting with Ambra1 may also contribute to senescence.

### Neural disease

FBXL20 (also known as SCRAPPER), a synapse-localized F-box protein, was proved to regulate neuronal synaptic tuning via the destruction of RIM1, which is required for synaptic vesicle release [145]. *Scrapper* knock-out mice displayed abnormal electrophysiological synaptic activity resulting from upregulation of RIM1. Moreover, FBXL20 is responsible for the ubiquitination and proteasomal degradation of Vps34, which controls intracellular vesicular processes, such as autophagy and endocytosis [75]. In light of the roles of endocytosis [146] and autophagy [147] in regulating synaptic development and plasticity, the control of Vps34 levels by SCF^FBXL20^ may provide an important regulatory mechanism for synaptic transmission and plasticity. Since many neural diseases are caused by excessive neurotransmitter release, future studies on FBXL20 might help elucidate their molecular pathogenesis.

Although a causal pathogenetic linkage between CRLs and neural disorders and diseases has not been established, the ubiquitin conjugates and/or inclusion bodies associated with ubiquitin have been discovered to be accumulated in a wide array of chronic neurodegenerative diseases [3]. In addition, NEDD8, one of the ubiquitin-like proteins, is also accumulated in ubiquitin-positive inclusions in various neurodegenerative disorders [148, 149]. Given that cullins are the best-characterized substrates of neddylation, the possible involvement of CRLs in the pathogenesis of neurodegeneration should not be neglected. In the mouse model of Huntington’s disease, inhibition of GPCR signaling by AMD3100, a selective GPCR antagonist, can induce autophagy by suppressing CRL3^ZBTB16^-mediated ATG14L degradation, leading to the expanded polyQ degradation and the preservation of neuronal functions [77]. Meanwhile, activated IRS2, a substrate of CRL5 [97], can induce autophagy in modified neural cell lines, used as models for Huntington’s disease, to enhance the clearance of polyQ proteins [150]. Thus, CRLs would play important roles in the pathogenesis of neurodegenerative diseases through their key substrates associated with autophagy.

### Cardiac disease

Atrogin-1, also known as FBXO32, is a skeletal and cardiac muscle-specific F-box protein [151]. Atrogin-1 was demonstrated as a critical player in skeletal muscle atrophy programs, and it is tightly regulated at the transcriptional level by FoxO factors [152]. Meanwhile, Atrogin-1 also induces the transcriptional activity of FoxO1/FoxO3 dependent on their K63-linked polyubiquitination mediated by SCF^Atrogin-1^. These findings were confirmed in *Atrogin*-*1* transgenic and knock-out mouse models, strongly indicating its crucial role in the inhibition of cardiac hypertrophy [123]. Moreover, accumulating data have proved that the autophagic activity governed by FoxO factors at multiple steps significantly contributes to cardiac homeostasis and disease [153]. All these studies suggest CRLs affect cardiac remodeling through regulating autophagic flux, which was further demonstrated in vivo. In *Atrogin*-*1* knock-out mice, Atrogin-1 depletion causes cardiomyopathy and premature death resulting from impaired autophagy [154]. Mechanistically, SCF^Atrogin-1^ promotes the ubiquitination and degradation of CHMP2B, which is part of an endosomal sorting complex required for autophagy [155]. Atrogin-1 deficiency failed to destroy CHMP2B, resulting in sequential serious consequences: autophagy impairment, protein aggregation, activation of unfolded protein response (UPR) signaling pathways, and ultimately, cardiomyocyte apoptosis [154]. In addition, cardiomyocyte-restricted *Csn8* knock-out (CR-Csn8KO) mouse model also proved the important role of Atrogin-1 in cardiomyocyte necrosis and dilated cardiomyopathy via autophagy impairment caused by down-regulation of Rab7, which is indispensable for autophagosome-lysosome fusion [156]. The underlying molecular mechanisms could be that (1) Atrogin-1 is down-regulated in Csn8-null heart, which is consistent with the theory that CSN-mediated deneddylation stabilizes F-box proteins [157, 158]; (2) Atrogin-1 enhances the transactivation of FoxO1/FoxO3 by promoting their ubiquitination; (3) Rab7 is a target gene of FoxO factors [159]. Taken together, Atrogin-1 plays a vital role in maintaining the homeostasis of cardiac myocytes through regulating autophagic flux.

### Cancer

Given that CRLs play a fundamental role in regulating a wide range of biological processes, including signal transduction, gene transcription, DNA replication, cell cycle progression, and apoptosis among others, it is anticipated that deregulation of CRLs is related to uncontrolled cell proliferation, ultimately leading to cancer [23]. It is widely accepted that autophagy plays an important role in tumorigenesis, hence autophagy regulated by CRLs more or less contributes to cancer development. For instance, (1) RBX1 knock-down triggers protective autophagy. Blockage of autophagy pathway significantly enhances the inhibition of tumor cell growth induced by RBX1 knock-down [106]. Similarly, (2) MLN4924, a general inhibitor of CRLs, also triggers a protective autophagy in many human cancer cell lines through mTORC1 inhibition resulting from the accumulation of DEPTOR and HIF1α, two well-known substrates of CRLs. Thus, autophagy inhibitors remarkably increase the apoptosis induced by MLN4924 [105]. Nevertheless, (3) *Rbx2* deletion in the skin inhibits autophagy and oncogene-activated senescence induced by Kras^G12D^, and consequently, promotes skin papillomagenesis. Thus Rbx2 acts as a skin-specific tumor suppressor by promoting autophagy via targeting its substrates: Erbin and Nrf2 [102].

## Conclusions and future perspectives

In summary, autophagy and UPS are crucial in the maintenance of cellular homeostasis, hence both of them need to be precisely orchestrated. CRLs, the largest E3 ubiquitin ligase family, mediate the degradation or activity alteration of many components and regulators in the autophagy pathway to control autophagic activity. Meanwhile, autophagy also conversely affects the activity of CRLs and UPS. The cross-talk between CRLs and autophagy deserves further intensive investigation to elucidate how the dysfunction of CRLs and autophagy contributes to the development of various human disorders, such as neural diseases, cardiac diseases, and cancer, which should provide new insights into drug discovery targeting CRLs and autophagy. In consideration of the facts that (1) CRLs are being validated as promising anti-cancer targets; (2) MLN4924, a small molecule indirect inhibitor of CRLs, which is currently in several Phase I clinical trials against a number of human malignancies, induces protective autophagy; (3) the inhibitors of autophagy significantly sensitize cancer cells, particularly resistant cancer cells, to MLN4924 treatment, future studies focused on CRLs and autophagy would eventually benefit human anti-cancer therapy.

Although some associations of CRLs and autophagy have been clarified, many fundamental questions still remain to be addressed: (1) what are other components of autophagy machinery and regulators associated with autophagy as the substrates of CRLs? (2) How does autophagy regulate the activity of CRLs and UPS? (3) Are the findings in cell culture settings consistent to those in physiological settings (knock-out/knock-in mouse models and patient samples)? (4) What is the function of deubiquitination in autophagy? Some recent studies have shown that deubiquitinases, enzymes catalyzing a reverse process for protein ubiquitination, also play a regulatory role in autophagy, such as USP36 and A20 [79, 160]. The answers to these fundamental questions would certainly uncover the precise roles of CRLs in the regulation of autophagy and autophagy-associated diseases, and provide molecular basis for rational drug design by targeting CRLs and autophagy.

## Abbreviations

Ambra1: activating molecule in Beclin-1-regulated autophagy
AMPK: AMP-activated protein kinase
ATF4: activating transcription factor 4
ATG: autophagy related gene
Bcl-2: B-cell lymphoma 2
BTB: bric-a-brac, tramtrack, broad-complex domain
β-TrCP: β-transducin repeat-containing protein
CAND1: cullin-associated and neddylation-dissociated 1
CHMP2B: charged multivesicular body protein 2B
CMA: chaperone-mediated autophagy
CRLs: cullin-RING ligases
CSN: COP9 signalosome complex
DAPK: death associated protein kinase
DCAF: DDB1-CUL4 associated factor
DDB1: DNA damage-binding protein 1
DEPTOR: DEP domain containing mTOR-interacting protein
DFCP1: double FYVE-containing protein 1
FBW: F-box and WD-40 domain protein
FBXL: F-box and leucine-rich repeat protein
FBXO: F-box only protein
HECT: homologous to E6-AP C-terminus
HIF1: hypoxia-inducible factor 1
IKK: inhibitor of κB kinase
IRS1: insulin receptor substrate 1
Keap1: kelch-like ECH-associated protein 1
KLHL20: kelch-like family member 20
LC3: microtubule-associated protein light chain 3
mTOR: mammalian target of rapamycin
NAE: NEDD8-activating enzyme
NEDD8: neural precursor cell expressed, developmentally down-regulated 8
NF1: neurofibromatosis type 1
NRF2: NF-E2 related factor 2
PDCD4: programmed cell death 4
PHLPP1: PH domain and leucine rich repeat protein phosphatase 1
PI3K: phosphatidylinositol-3-kinase
polyQ: polyglutamine
RBX1/2: RING box protein 1/2
REDD1: regulated in development and DNA damage responses 1
RIM1: Rab3-interacting molecule 1
RING: really interesting new gene
RNF5: RING finger protein 5
ROC1/2: regulator of cullins 1/2
Rubicon: RUN domain protein as Beclin-1 interacting and cysteine-rich containing
S6K1: ribosomal S6 kinase 1
SAG: sensitive to apoptosis gene
SCF: SKP1, cullin and F-box protein
SKP1/2: S-phase kinase-associated protein 1/2
SOCS: suppressors of cytokine signaling
SQSTM1: sequestosome 1
TRAF6: TNF-receptor-associated factor 6
TSC1/2: tuberous sclerosis 1/2
UPR: unfolded protein response
UPS: ubiquitin-proteasomal system
USP: ubiquitin specific protease
UVRAG: UV radiation resistance associated gene
VHL: Von Hippel–Lindau
WIPI: WD-repeat domain protein interacting with phosphoinositides
ZBTB16: zinc finger and BTB domain containing 16
